# The transcriptomic response of *Hyphantria cunea* (Drury) to the infection of *Serratia marcescens* Bizio based on full-length SMRT transcriptome sequencing

**DOI:** 10.3389/fcimb.2023.1093432

**Published:** 2023-02-16

**Authors:** Ling Zhang, Xinyi Tang, Zhiqiang Wang, Fang Tang

**Affiliations:** ^1^ Co-Innovation Center for Sustainable Forestry in Southern China, Nanjing Forestry University, Nanjing, China; ^2^ College of Forestry, Nanjing Forestry University, Nanjing, China

**Keywords:** *Hyphantria cunea* (Drury), full-length transcriptome, SMRT sequencing, *Serratia marcescens* Bizio, transcriptomic response

## Abstract

*Hyphantria cunea* (Drury) is a globally important forest pest. We found that the *Serratia marcescens* Bizio strain SM1 had insecticidal activity against *H. cunea*, but the transcriptomic response of *H. cunea* to SM1 were not clear. Therefore, we performed full-length sequencing of the transcriptomes of *H. cunea* larvae infected with SM1 and the control group. A total of 1,183 differentially expressed genes (DEGs) were identified by comparing the group infected with SM1 and the control group, including 554 downregulated genes and 629 upregulated genes. We found many downregulated genes in metabolic pathways. Furthermore, some of these downregulated genes were involved in cellular immunity, melanization, and detoxification enzymes, which showed that SM1 weakened *H. cunea* immunity. In addition, genes in the juvenile hormone synthesis pathway were upregulated, which was detrimental to the survival of *H. cunea*. This research analyzed the transcriptomic response of *H. cunea* to SM1 by high-throughput full-length transcriptome sequencing. The results provide useful information to explore the relationship between *S. marcescens* and *H. cunea*, and theoretical support for the application of *S. marcescens* and the control of *H. cunea* in the future.

## 1 Introduction

*Hyphantria cunea* (Drury) originated in North America, was introduced into east Asia and central Europe in the early 1940s, and then spread to Eurasia. At present, it is found in more than 30 countries and is an important forest pest worldwide (Ge et al., 2019). In 1979, it invaded the Dandong area of China’s Liaoning Province and gradually spread throughout China (Ji et al., 2003). Studies have shown that *H. cunea* has adapted to local climate conditions following its invasion and range expansion (Zhao et al., 2021). *H. cunea* has more than 600 host plants, including a variety of forestry and fruit trees, shrubs, herbs, and crops (Edosa et al., 2019). Due to its variety of host plants, large food intake, and strong reproductive and transmission ability, its invasion has seriously undermined the ecological environment, landscape, agriculture, forestry and many other aspects in China and caused great economic and environmental losses to China (Zhao, 2005). As an important pest control method, pathogenic microorganisms have been studied for their interactions with various pests (Xu et al., 2018; Bai et al., 2022). To control *H. cunea*, scientists have explored the use of various pathogenic microorganisms such as *Bacillus thuringiensis*, *Beauveria bassiana*, and *H. cunea* nucleopolyhedrovirus (HcNPV) (Zibaee et al., 2010; Aker & Tuncer, 2016; Sun et al., 2019).

*Serratia marcescens* Bizio (Enterobacterales: Yersiniaceae) can be used as a biocontrol bacterium to control some insect pests and plant pathogenic fungi (Nehme et al., 2007; Babashpour et al., 2012; Wang et al., 2013). Chen et al. (2001) isolated a strain of *S. marcescens* from dead *Helicoverpa armigera* (Hübner) and found that the strain had strong virulence on several insects, including *Spodoptera exigua* (Hübner), *Pieris rapae* (Linnaeus) and *H. armigera*. In addition, *S. marcescens* has also been found to be pathogenic to pests such as *Diaphorina citri* Kuwayama, *Phthorimaea operculella* (Zeller) and *Spodoptera litura* (Fabricius) (Aggarwal et al., 2017; Hu et al., 2018; Su et al., 2020). In our laboratory, we isolated a *S. marcescens* strain SM1 (hereafter refer to as SM1), and this strain had insecticidal activity against the larvae of *H. cunea*, indicating that SM1 has biocontrol potential against *H. cunea* (Feng et al., 2021).

We have previously showed that SM1 can induce strong immune responses of *H. cunea* by Solexa sequencing using an Illumina NovaSeq. Specifically, three signaling pathways, melanization and several cellular immune responses were activated, besides, several immune-related genes were also upregulated, including cytochrome P450s and uridine diphosphate-glycosyltransferases (Feng et al., 2021; Wang et al., 2021a). In addition to the induction of immune reactions, nonetheless, previous studies showed that many pathogenic bacteria can produce detrimental chemical toxins to affect a series of other genes expression. Thus, a comparative analysis of the transcriptomic response in *H. cunea* against SM1 using full-length single molecule real-time (SMRT) transcriptome sequencing may facilitate the understanding of the interaction between the pest and the bacterial pathogen. As a third-generation high-throughput sequencing technology, SMRT sequencing technology has been applied to transcriptome sequencing and analysis of many species, such as *Agasicles hygrophila* (Selman and Vogt), *Bactrocera dorsalis* (Hendel), *Odontotermes formosanus* (Shiraki), *Rhopalosiphum padi* (Linnaeus), *Rhynchophorus ferrugineus* (Olivier), and *Sogatella furcifera* (Horvath) (Jia et al., 2018; Chen et al., 2020; Feng et al., 2020; Yang et al., 2020; Ouyang et al., 2021; Wang et al., 2021b). Compared with the Sanger method and next-generation sequencing (NGS) technologies, SMRT sequencing technology has the advantages of being PCR-free, having a high speed, having long read lengths, and being capable of directly detecting epigenetic modifications, so it is very suitable for *de novo* genomic sequencing and high-quality assemblies of small genomes (Liu et al., 2015). In this study, the transcriptomic response on the metabolism, immunity and hormones of *H. cunea* to the infection of SM1 were analyzed using SMRT sequencing technology, which will provide the theoretical basis for the application of SM1 in the biocontrol of *H. cunea*.

## 2 Materials and methods

### 2.1 Insects and pathogenic bacteria

We collected *H. cunea* larvae in Huai’an, Jiangsu Province, China. In our laboratory, *H. cunea* larvae were fed with fresh poplar leaves in clear plastic boxes (20 cm×14 cm×10 cm) at an ambient temperature of 26 ± 1 °C with a light condition of 16 h light:8 h dark.

SM1 was isolated and stored in 25% glycerol at -80 °C.

### 2.2 Sample processing

SM1 was cultured on solid bacterial basal medium in the dark for 12 h at 30 °C to produce single colonies. A single colony of SM1 was proliferated with 50 mL seed culture medium at 200 r/min at 30 °C for 12 h. An appropriate amount of seed solution was added to 200 mL fermentation medium at 200 r/min at 30 °C for 36 h.

Healthy third instar *H. cunea* larvae were selected as experimental materials. Fresh poplar leaves of similar size were soaked in SM1 fermentation medium for 10-15 s, then two leaves with dry surface and 20 *H. cunea* larvae were placed into the triangular bottle (treatment group, SM_HC); leaves were soaked in sterile fermentation medium as the control group (CK). Live *H. cunea* larvae treated for 70 h were collected and stored at -80 °C for transcriptome sequencing. The test was repeated three times.

### 2.3 RNA sample preparation

We uesd the TRIzol method to extract total RNA from the samples. A Nanodrop 2000 spectrophotometer was used to determine the concentration and purity, and the integrity of RNA was detected by using agarose gel electrophoresis.

### 2.4 Library preparation and SMRT sequencing

The main process of library construction was as follows: The Clonetech SMARTerTM PCR cDNA Synthesis Kit was used to synthesize full-length cDNA of mRNA. Primer with Oligo dT was used for A-T base pairing with the polyA tail at the 3’ terminal of mRNA as primer for reverse synthesis of cDNA, and primer was added to the terminal of full-length cDNA synthesized in reverse. Full-length cDNA was obtained using PCR, PB magnetic beads were used to purify the amplified full-length cDNA, and small fragments of cDNA less than 1 kb were removed. The terminus of the full-length cDNA was repaired and connected to the SMRT dumbbell adapter. The fragments that were not connected to the adaptor were digested by exonuclease. PB magnetic beads were used to purify the fragments, and the sequencing library was obtained. After library construction, Qubit 3.0 was used for accurate quantification, and Agilent 2100 was used to detect the size of the library. After qualified detection, a PacBio sequencer was used to perform full-length transcriptome sequencing. The raw sequence generated by the PacBio sequencer totaled 86.4 Gbp and was deposited into the NCBI Sequence Read Archive (SRA) with accession number SRR22263802.

### 2.5 Sequel data output and quality control

SMRTlink software was used to preprocess the raw sequencing data based on the following main parameters: minimum predicted accuracy = 0.99, minimum number of passes = 3, maximum subread length = 15,000, minimum subread length = 50, and full-length transcript sequences were obtained by using the Iso-Seq analysis process. Subreads were obtained by splitting the single-molecule polymerase reads, and the subreads obtained from the same polymerase reads were self-corrected to form circular consensus sequences (CCS). CCSs were classified and full-length non-concatemer (FLNC) sequences were found by detecting chimeric sequences, primer sequences and 3′ poly-A sequences. The FLNC sequences were clustered, and redundancy was eliminated by the iterative clustering and error correction (ICE) tool of SMRTlink software and then further corrected by the arrow algorithm in SMRTlink, by which the obtained sequences were considered polished transcripts. Cd-hit software was used to cluster and remove redundancy (Li and Godzik, 2006), and the final full-length transcript sequence was used for subsequent analysis.

### 2.6 Functional annotation of transcripts

The basic functional annotations of full-length transcripts include NCBI nonredundant protein sequences (NR), Gene Ontology (GO), Kyoto Encyclopedia of Genes and Genomes (KEGG), Clusters of Orthologous Groups/Eukaryotic Orthologous Groups (COG/KOG) and Swiss-Prot Protein Sequence Database (Swiss-Prot) annotations. Isoform sequences were aligned to the NR, KEGG, COG/KOG and Swiss-Prot databases by diamond blastx, and protein IDs with high sequence similarity were obtained to gain the protein functional annotation information of the isoforms (Buchfink et al., 2015). According to the annotation results of five databases, the annotation status of all transcripts was statistically summarized, and the transcripts with ambiguous or contradictory annotations in the initial analysis were further verified by the BLAST function of the NCBI website (https://www.ncbi.nlm.nih.gov/).

### 2.7 Digital gene expression library preparation and analysis

The isoforms that had redundancy removed were used as the reference transcriptome sequences, and bowtie2 software was used to compare the clean reads of each sample with the references (Langmead, 2010). The comparison results of bowtie2 were counted by using RSEM, the number of reads from each sample to each transcript was obtained, and fragments per kilobase per million bases (FPKM) conversion was performed (Li and Dewey, 2011). The paired-end reads from the same fragment were counted as one fragment, and the expression levels of isoforms and transcripts were obtained. DESeq2 was used for differential expression analysis (Love et al., 2014). The threshold of the P-value in multiple tests was determined by using the false discovery rate (FDR) method. We set a threshold of FDR < 0.05 and an absolute value of log_2_fold change (FC) > 1 or log_2_FC < -1 to judge the significance of the difference in gene expression. Then, the genes expressed at different levels in the samples were further annotated by performing GO enrichment analysis and KEGG pathway enrichment analysis.

## 3 Results

### 3.1 Overview of the full-length transcriptome database

#### 3.1.1 Sequencing data output and transcript clustering analysis

A total of 533,521 polymerase reads were generated by using PacBio SMRT sequencing technology. After removing the adapter sequences of the polymerase reads, the remaining sequence fragments are called subreads. 45.92 Gbp of subreads were obtained after preprocessing. CCSs are sequences with low error rates obtained by correcting sequencing errors among multiple sequencing results. In total, 403,952 CCSs were obtained. By classifying CCSs, a total of 333,359 FLNC sequences were identified. By clustering and correcting FLNC sequences using SMRTlink software, 18,322 polished transcripts were assembled. Using cd-hit software to cluster and eliminate redundancy, a total of 17,483 isoforms were assembled for further study. The above data details can be seen in **Table 1**.

**Table 1:** Statistics of sequencing data and transcript clustering data.

Data type	Total bases(bp)	Total number	Minimum length	Average length	Maximum length	N50	Average_accuracy	Average_passes
polymer read	49.9G	533,521	51	93,532	386,474	179,304	/	/
subread	45.92G	49,304,066	51	932	280,286	1,011	/	/
CCS	468,059,677	403,952	52	1,159	10,138	1,316	0.99946	102
FLNC	339,891,439	333,359	50	1,020	7,327	1,165	/	/
polished transcript	19,686,284	18,322	64	1,075	6,357	1,298	/	/
non redundant isoform	18,648,983	17,483	64	1,067	6,355	1,296	/	/

#### 3.1.2 Functional annotation of transcripts

In total, 11,912 transcripts were annotated in the NR database; 3,581 transcripts were annotated in the GO database; 6,580 transcripts were annotated in the KEGG database; 7,855 transcripts were annotated in the KOG database; and 8,761 transcripts were annotated in the SwissProt database. Additionally, 2,467 transcripts were annotated in all of these databases, and 5,564 transcripts were not annotated in any of these databases.

**Figure 1:**
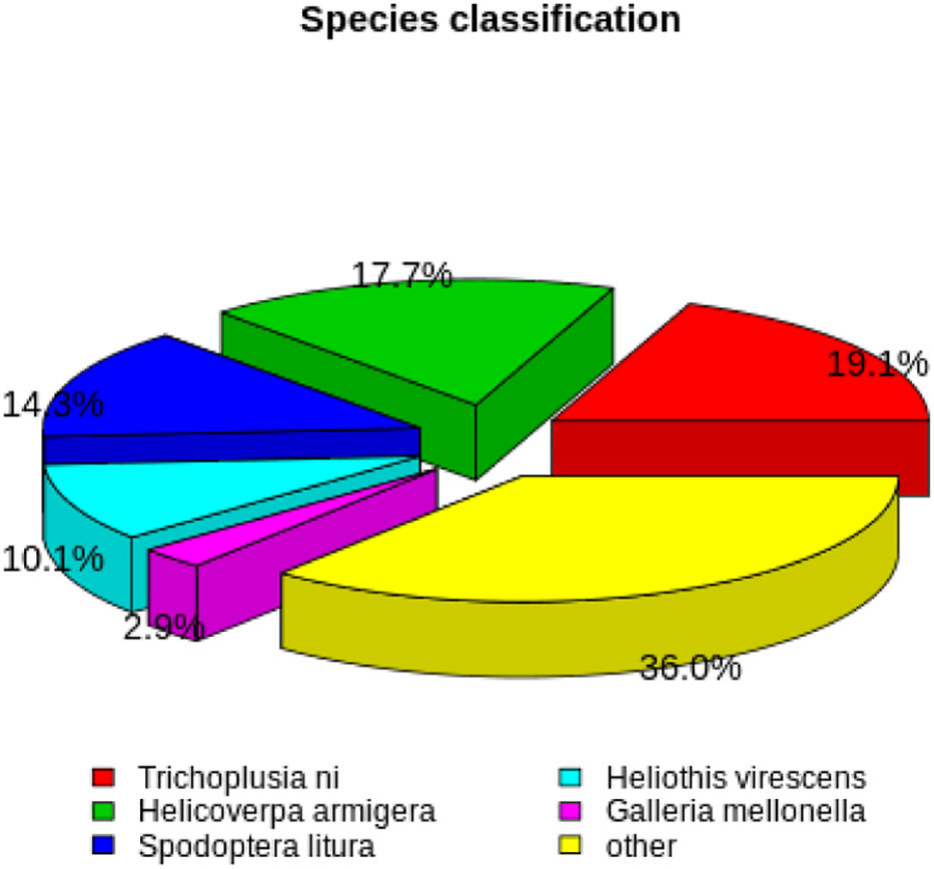
Homologous species distribution of *H. cunea* annotated in the NR database.

Based on NR annotations, 19.1% of the *H. cunea* sequences were aligned to *Trichoplusia ni* (Hübner), followed by *H. armigera* (17.7%), *S. litura* (14.3%), *Heliothis virescens* (Fabricius) (10.1%), *Galleria mellonella* (Linnaeus) (2.9%), and others (36.0%) (**Figure 1**).

**Figure 2:**
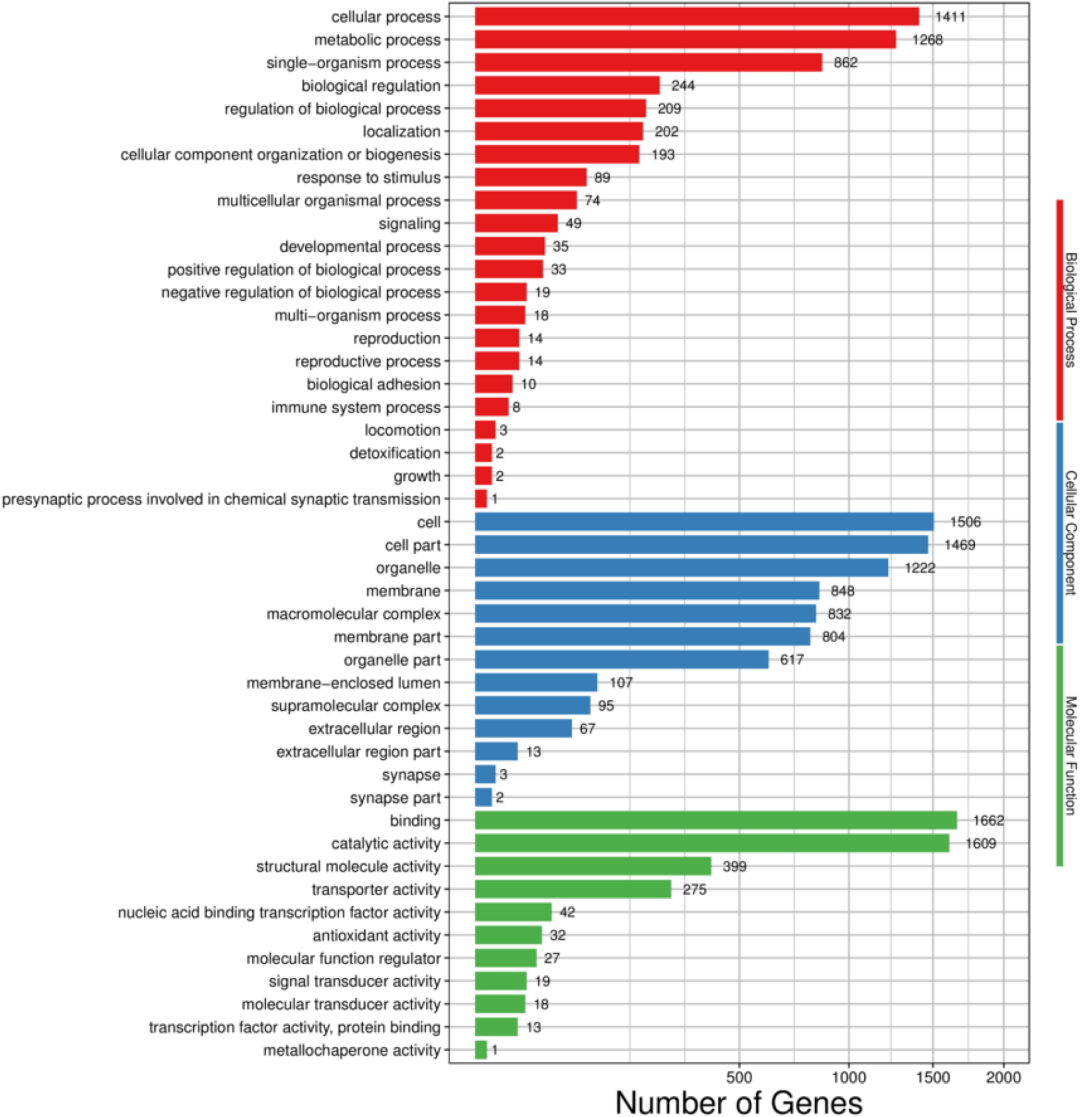
GO functional classifcations of *H. cunea* transcripts. Red, blue and green represent biological process, cellular component and molecular function, respectively. The x-axis represents the number of transcripts; the y-axis represents GO categories.

Based on GO annotations, the transcriptome of *H. cunea* was distributed to 46 GO terms of the three main functional processes. In the biological process (BP) category, cellular process (1411) and metabolic process (1268) had the most transcripts. With respect to the cellular component (CC) category, the most abundant terms were cell (1506) and cell part (1469), followed by organelle (1222). Regarding the molecular function (MF) category, binding (1662) and catalytic activity (1609) were the subcategories with the most transcripts (**Figure 2**).

**Figure 3:**
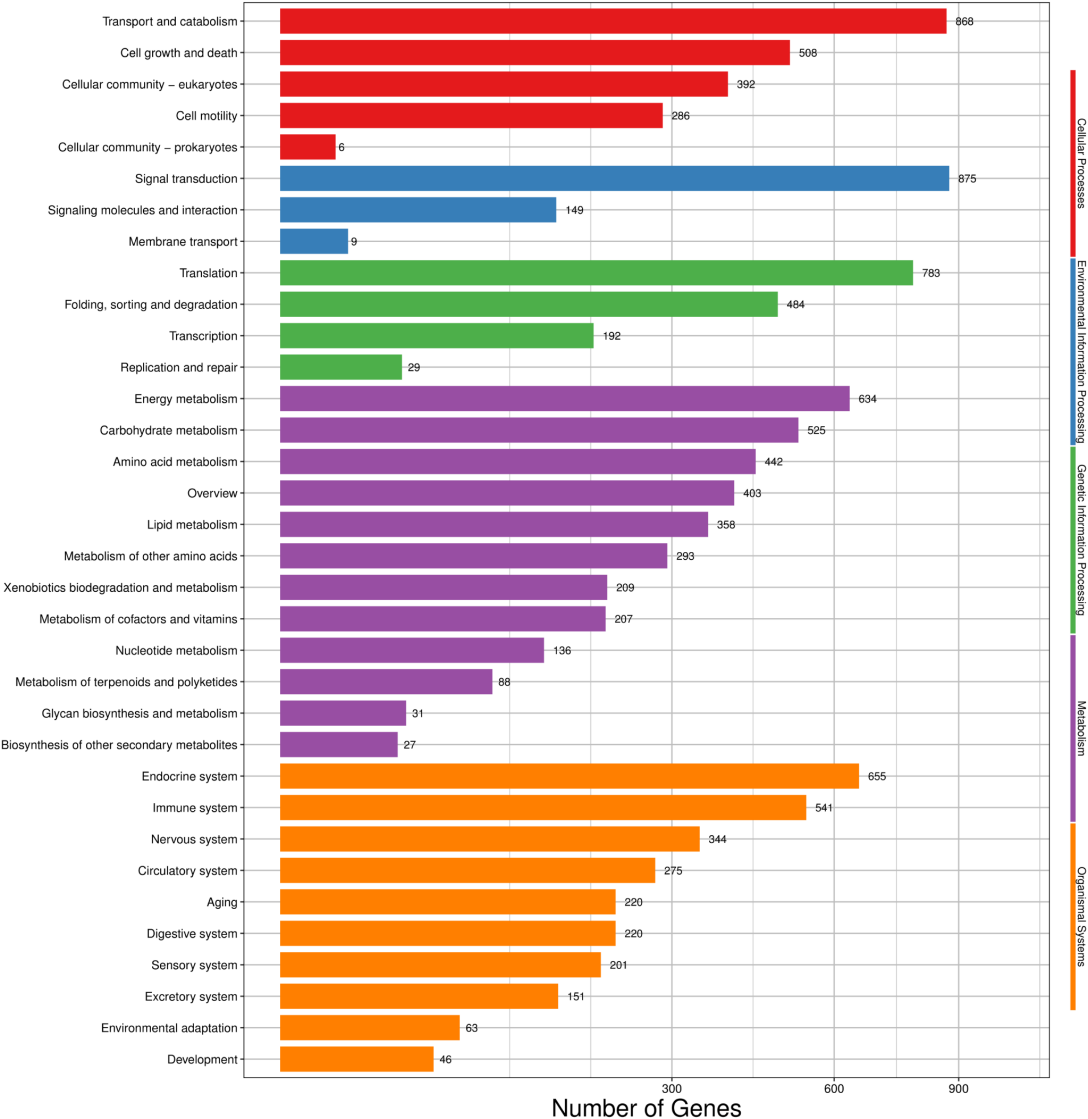
KEGG classification of *H. cunea* transcripts. Red, blue, green, purple and orange represent cellular processes, environmental information processing, genetic information processing, metabolism and organismal systems, respectively. The x-axis represents the number of transcripts; the y-axis represents KEGG pathway categories.

A total of 6,580 transcripts were annotated into 275 KEGG pathways. These KEGG pathways can be divided into five large branches, including cellular processes, environmental information processing, genetic information processing, metabolism and organismal systems, and further divided into 34 smaller branches. For cellular processes, transport and catabolism (868) had the largest numbers of transcripts. For environmental information processing, signal transduction (875) was the main subcategory. For genetic information processing, translation (783) was the most abundant term. For metabolism, energy metabolism (634) was the term with the largest numbers of transcripts. For organismal systems, the largest number of transcripts was assigned to the endocrine system (655) (**Figure 3**).

**Figure 4:**
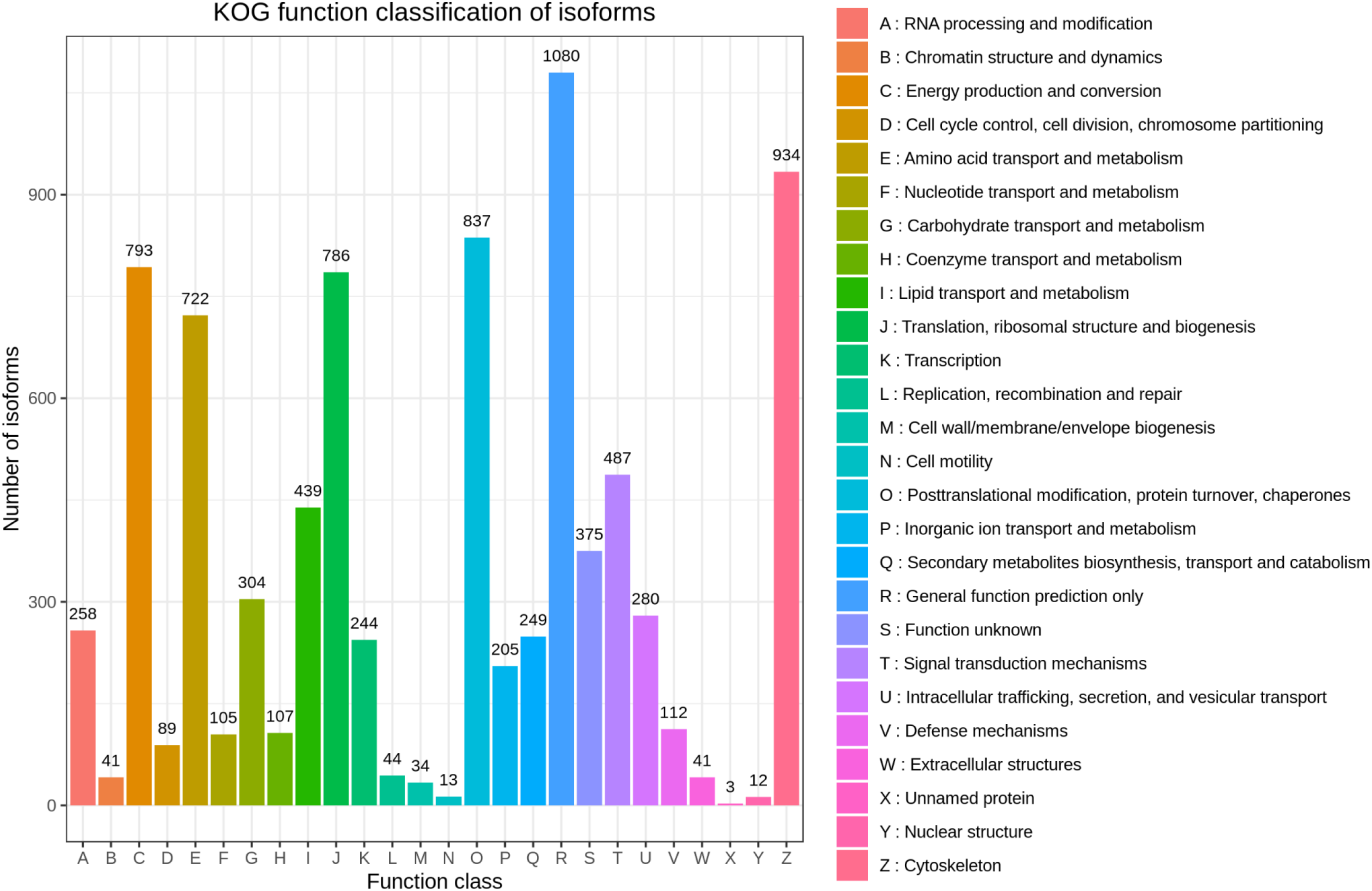
KOG annotation of *H. cunea* transcripts. The x-axis represents KOG categories; the y-axis represents the number of transcripts.

Based on the KOG database, 7,855 transcripts were annotated and grouped into 26 KOG groups. General function prediction only (1080) was the largest group in the 26 KOG groups, followed by Cytoskeleton (934) and Posttranslational modification, protein turnover, chaperones (837) (**Figure 4**).

#### 3.1.3 DEGs in *H. cunea* in response to SM1 infection

**Figure 5:**
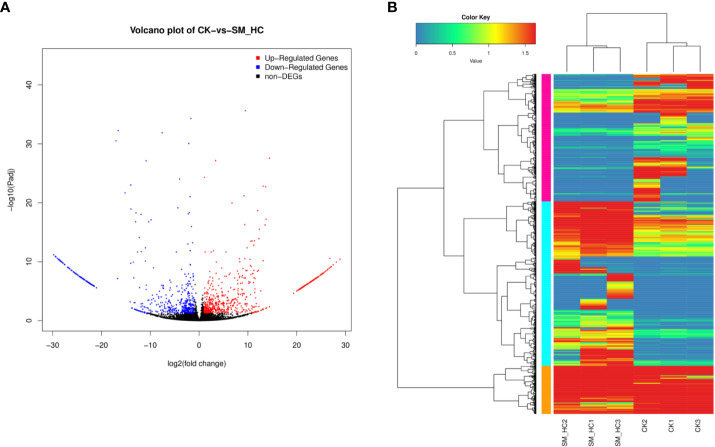
Overview of DEGs. **(A)** Comparison of DEGs between the CK library and SM_HC library. Red spots represent signifcantly upregulated genes and blue spots represent significantly downregulated genes. Black spots indicate no significant diferences in gene expression. **(B)** Heatmaps illustrating diferences in normalized log signal intensity for the identified *H cunea* genes. *S. marcescens* treatment groups were labeled as SM_HC1, SM_HC2 and SM_HC3, and control groups were labeled as CK1, CK2 and CK3. Red indicates genes expressed at high levels and blue indicates genes expressed at low levels. The colors from blue to red indicate gradually increasing expression.

We attempted to identify the upregulated and downregulated DEGs in *H. cunea* infected with SM1 by using PacBio Sequel sequencing to investigate the transcriptomic response of *H. cunea*. To improve the accuracy of expression measurements, we combined the data from three biological replicates. Using the combined data, FPKM values were computed, and the results were compared between the replicate SM_HC and CK groups. When FDR < 0.05 and log_2_FC > 1 or log_2_FC < -1, DEGs were considered to be significantly different between the SM_HC and CK groups (**Figure 5A**). In total, 1,183 DEGs were identified, including 629 upregulated DEGs and 554 downregulated DEGs (**Figure 5B**).

**Figure 6:**
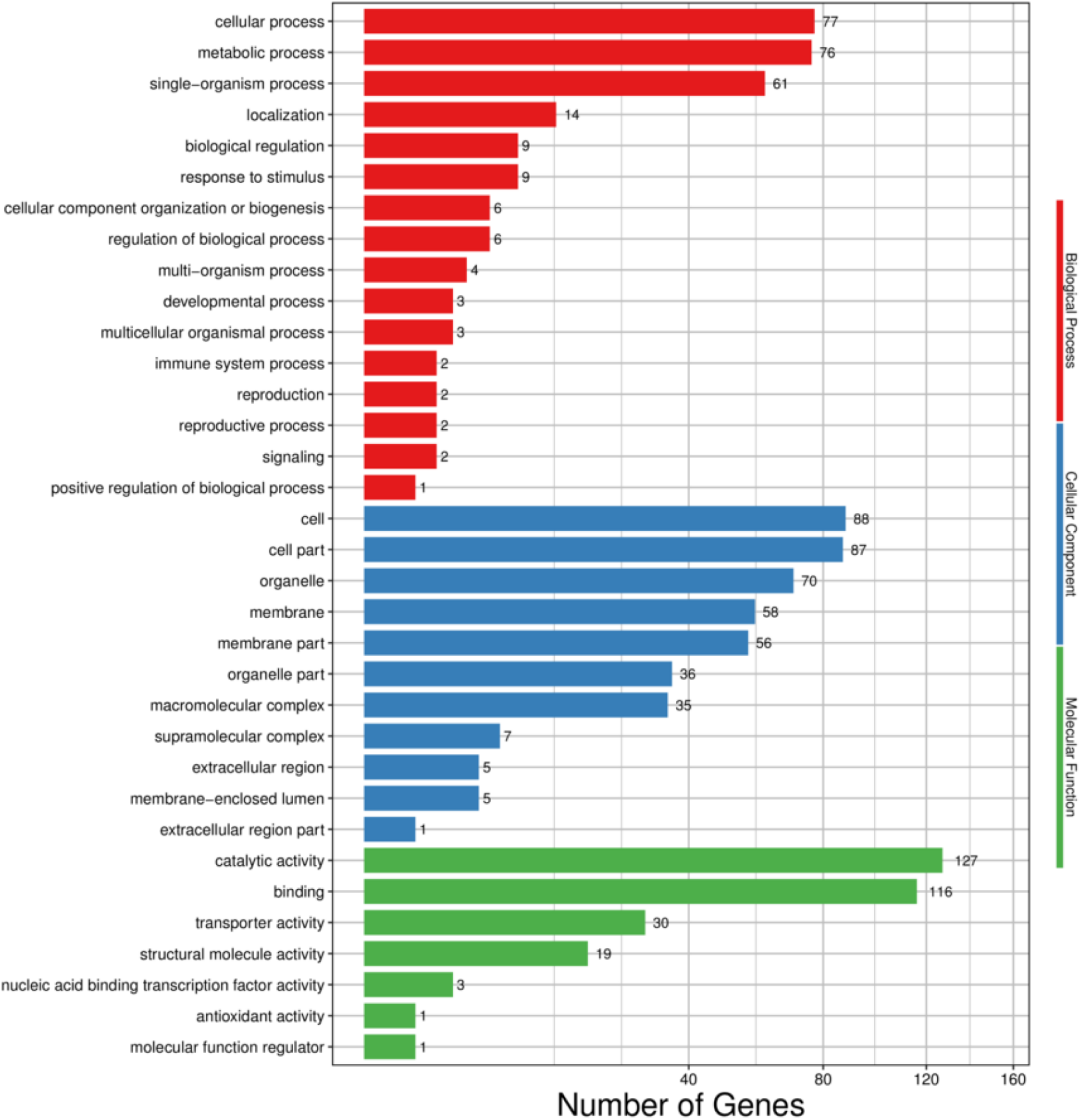
GO functional classifcation of DEGs in *H. cunea*. Red, blue and green represent biological process, cellular component and molecular function. The x-axis represents the number of transcripts; the y-axis represents GO categories.

Based on GO enrichment analysis, the functions of the 244 DEGs in *H. cunea* were classified into 34 groups of three main categories. In the BP category, cellular process (77) and metabolic process (76) were the most abundant terms, followed by single-organism process (61). With regard to the CC category, cell (88), cell part (87) and organelle (70) were highly enriched. In terms of the MF category, the main subcategories were catalytic activity (127) and binding (116) (**Figure 6**).

DEGs were mapped to typical KEGG pathways, identifying biological pathways that respond to SM1 treatment, and a total of 319 DEGs were distributed into 170 KEGG pathways (**Supplementary Table S1**). The pathways were considered highly enriched when the P-values were <0.05 (**Table 2**). Based on DEG enrichment analysis, the highly enriched pathway with the most unigenes was phagosome (37), followed by carbon metabolism (31), apoptosis (28) and cardiac muscle contraction (25).

**Table 2:** Highly enriched KEGG pathways of DEGs in the *H. cunea* transcriptome.

#	KEGG Pathway	DEGs with pathway annotation (Gene Number)	All genes with pathway annotation (Background number)	P-Value	Pathway ID
1	Phagosome	37	402	0.01820028	ko04145
2	Carbon metabolism	31	281	0.002350482	ko01200
3	Apoptosis	28	309	0.04388318	ko04210
4	Cardiac muscle contraction	25	215	0.003031961	ko04260
5	Lysosome	23	187	0.002136078	ko04142
6	Glutathione metabolism	20	141	0.000710598	ko00480
7	Protein digestion and absorption	19	118	0.000186526	ko04974
8	Valine, leucine and isoleucine degradation	17	136	0.006629671	ko00280
9	Neuroactive ligand-receptor interaction	17	116	0.00121487	ko04080
10	Fatty acid degradation	16	142	0.02110153	ko00071
11	PPAR signaling pathway	15	139	0.03517465	ko03320
12	Longevity regulating pathway - multiple species	15	121	0.01122485	ko04213
13	Renin-angiotensin system	15	62	0.000006558809	ko04614
14	Arginine and proline metabolism	14	109	0.01043517	ko00330
15	beta-Alanine metabolism	14	105	0.007559999	ko00410
16	Lysine degradation	13	110	0.02517429	ko00310
17	Fatty acid metabolism	12	99	0.02582494	ko01212
18	Hematopoietic cell lineage	12	43	0.00001156149	ko04640
19	Two-component system	11	53	0.000479384	ko02020
20	Calcium signaling pathway	10	82	0.03845356	ko04020
21	Glycine, serine and threonine metabolism	9	72	0.04208052	ko00260
22	RNA degradation	9	71	0.03895367	ko03018
23	Histidine metabolism	8	57	0.02954309	ko00340
24	Propanoate metabolism	8	54	0.02200662	ko00640
25	Limonene and pinene degradation	7	52	0.04920007	ko00903
26	2-Oxocarboxylic acid metabolism	7	51	0.0449744	ko01210
27	Starch and sucrose metabolism	7	38	0.01006243	ko00500
28	Phenylalanine metabolism	6	24	0.003556592	ko00360
29	Phenylalanine, tyrosine and tryptophan biosynthesis	5	21	0.009609174	ko00400
30	Aminobenzoate degradation	4	21	0.04338899	ko00627
31	Steroid biosynthesis	3	13	0.04766836	ko00100
32	GABAergic synapse	3	12	0.03847072	ko04727

### 3.2 Transcriptomic response of *H. cunea* to SM1 infection

#### 3.2.1 SM1 suppressed the expression of metabolism-related genes of *H. cunea*

**Figure 7:**
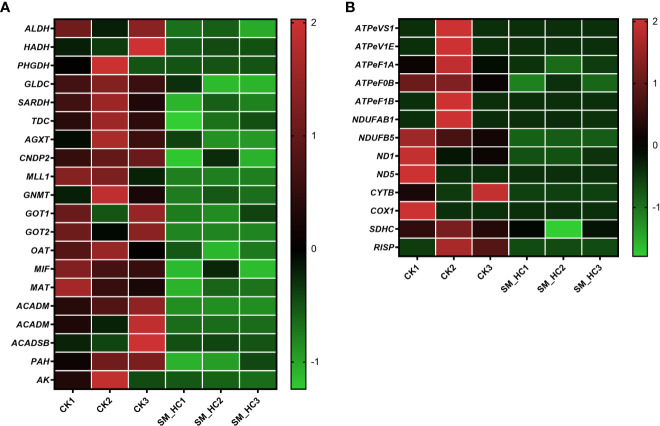
Heatmap analysis of downregulation genes in the metabolic pathways in *H cunea* infected by SM1. The control groups were labeled as CK1, CK2 and CK3. *H cunea* groups infected with SM1 were labeled as SM_HC1, SM_HC2 and SM_HC3. **(A)** Amino acid metabolism. **(B)** Oxidative phosphorylation.

Metabolism of organisms is an important link for maintaining normal physiological activities. We searched the KEGG database and filtered out genes related to metabolic pathways of *H. cunea*, which could be divided into energy metabolism, metabolism of cofactors and vitamins, amino acid metabolism, metabolism of terpenoids and polyketides, nucleotide metabolism, xenobiotic biodegradation and metabolism, carbohydrate metabolism, and lipid metabolism. The results indicated that SM1 infection regulated a large number of genes in *H. cunea*, and many of them were downregulated. For example, a total of 20 genes were downregulated in amino acid metabolism (**Figure 7A**) and 13 genes were downregulated in the oxidative phosphorylation pathway (**Figure 7B**) (**Supplementary Table S2**). In this study, downregulated genes in amino acid metabolism were distributed in histidine-, glycine-, serine-, threonine-, alanine-, aspartate-, glutamate-, tryptophan-, tyrosine-, cysteine-, methionine-, valine-, leucine-, isoleucine-, lysine-, phenylalanine-, arginine- and proline-related metabolic pathways. Oxidative phosphorylation is an important part of biological energy metabolism. In our results, the downregulated genes in the oxidative phosphorylation pathway accounted for most of the total downregulated genes related to energy metabolism. A total of 16 genes were downregulated in energy metabolism, of which 13 genes were located in the oxidative phosphorylation pathway. The 13 genes included NADH dehydrogenase (*NDUFAB1*, *NDUFB5*, *ND1* and *ND5*), cytochrome b (*CYTB*), cytochrome c oxidase (*COX1*), succinate dehydrogenase cytochrome b560 subunit (*SDHC*), Rieske iron-sulfur protein (*RISP*) and ATPase (*ATPeVS1*, *ATPeV1E*, *ATPeF1A*, *ATPeF0B* and *ATPeF1B*). In addition, V-type proton ATPase (*ATPeVS1* and *ATPeV1E*) genes appeared not only in the oxidative phosphorylation pathway but also in cellular immune-related pathways. In summary, SM1 infection led to downregulation of some metabolism-related genes in *H. cunea*.

#### 3.2.2 SM1 suppressed the immunity of *H. cunea*

##### 3.2.2.1 SM1 inhibited the expression of cellular immune-related genes in *H. cunea*

**Figure 8:**
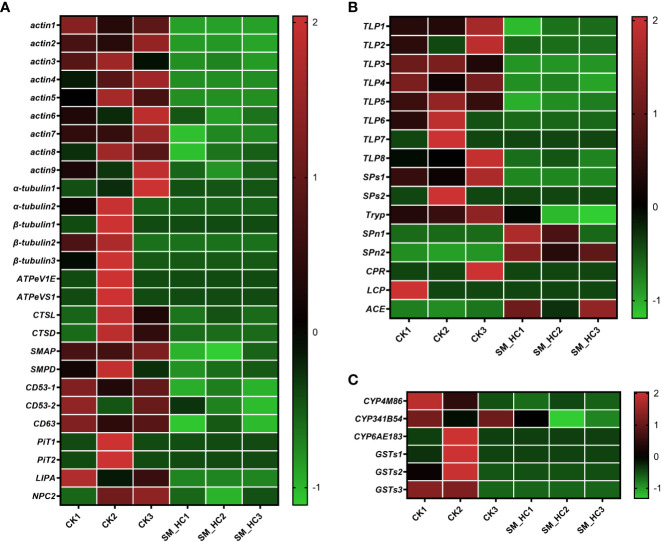
Heatmap analysis of downregulation immune-related genes in *H cunea* infected by SM1. The control groups were labeled as CK1, CK2 and CK3. *H cunea* groups infected with SM1 were labeled as SM_HC1, SM_HC2 and SM_HC3. **(A)** Cellular immune. **(B)** Melanization. **(C)** Detoxification enzyme.

We detected many cellular immune-related genes, including genes in the apoptosis, lysosome, autophagy, endocytosis and phagosome pathways. Some of these genes were downregulated in *H. cunea* infected with SM1 compared with uninfected *H. cunea* (**Figure 8A** and **Supplementary Table S2**). Actin and tubulin are two kinds of proteins associated with the cytoskeleton. In *H. cunea* larvae infected with SM1, 9 actin genes, 2 α-tubulin genes and 3 β-tubulin genes were downregulated. Actin and α-tubulin genes appear in the apoptosis pathway and phagosome pathway, and β-tubulin is involved in the phagosome pathway. V-type proton ATPase subunit E (*ATPeV1E*) and V-type proton ATPase subunit S1 (*ATPeVS1*) each had one downregulated gene. These 2 genes are present in the phagosome pathway, while *ATPeVS1* gene is also present in the lysosome pathway. Both cathepsin L (*CTSL*) and cathepsin D (*CTSD*) are involved in apoptosis, lysosome and autophagy pathways, and cathepsin L is also involved in phagocytosis. Each of the 2 cathepsins had one downregulated gene. In addition, 1 sphingomyelin phosphodiesterase (*SMPD*) gene, 2 CD53 antigen (*CD53-1* and *CD53-2*) genes, 1 *CD63* antigen (*CD63*) gene and 1 Niemann-Pick C2 protein (*NPC2*) gene were downregulated in the lysosome pathway, and 1 gene of stromal membrane-associated protein (*SMAP*) was downregulated in the endocytosis pathway. In short, SM1 infection downregulated many genes related to cellular immunity in *H. cunea*, which destroyed the cellular immune response.

##### 3.2.2.2 SM1 inhibited the melanization in *H. cunea*

Melanization is an important defense against pathogens in many arthropods. We searched the transcriptome data for genes associated with melanization. The results showed that SM1 infection weakened melanization of *H. cunea* larvae by regulating some genes (**Figure 8B** and **Supplementary Table S2**). As part of the protease cascade of melanization, serine proteases (SPs) promote the melanization process. In our study, two SPs (*SPs1* and *SPs2*) genes were downregulated in *H. cunea* larvae infected with SM1. Trypsin, a class of serine proteases, also participates in and promotes melanization. In our data, 8 trypsin-like protein (TLP) genes and 1 trypsin-like serine protease (Tryp)-related gene were downregulated by SM1. As a negative regulator of the serine protease cascade pathway, two serine protease inhibitor (*SPn1* and *SPn2*) genes were upregulated. Cuticular protein is a component of the melanic color pattern, and 2 genes related to cuticular protein (*CPR* and *LCP*) were downregulated in *H. cunea* larvae infected with SM1. In addition, as negative regulator of melanization, angiotensin converting enzyme (*ACE*) was upregulated after SM1 infection. In conclusion, SM1 infection inhibited the melanization in *H. cunea*.

##### 3.2.2.3 SM1 caused the downregulation of detoxification enzyme genes in *H. cunea*

In insect detoxification, P450s (CYPs) and glutathione S-transferases (GSTs) both play important roles. Our results showed that the expression of 3 *CYPs* and 3 *GSTs* were downregulated in *H. cunea* infected with SM1 (**Figure 8C** and **Supplementary Table S2**). After sequence alignment and naming by the cytochrome P450 nomenclature committee, these three *CYPs* were determined to be *CYP4M86*, *CYP341B54* and *CYP6AE183*, respectively. The three downregulated *GSTs* all belonged to the sigma class of cytosolic GSTs (*GSTs1*, *GSTs2* and *GSTs3*). In this study, SM1 infection caused the downregulation of some detoxification enzyme genes in *H. cunea*.

#### 3.2.3 SM1 induced juvenile hormone synthesis-related genes in *H. cunea*

**Figure 9:**
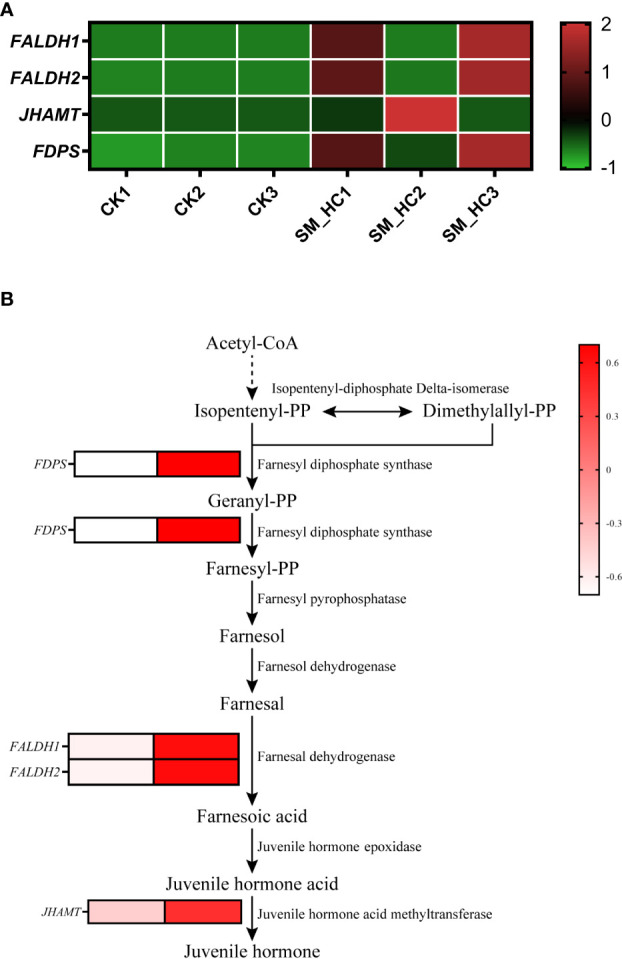
Changes in JH synthesis in *H cunea* infected by SM1. **(A)** Heatmap analysis of upregulation genes in JH synthesis using transcriptome data. The control groups were labeled as CK1, CK2 and CK3. *H cunea* groups infected with SM1 were labeled as SM_HC1, SM_HC2 and SM_HC3. **(B)** Diagram of genes related to JH synthesis in *H cunea* infected by SM1.

Juvenile hormone (JH) is an important hormone regulating insect growth, development and reproduction. In our study, after SM1 infection, there were 5 DEGs in the JH synthetic pathway of *H. cunea*, and 4 genes were upregulated (**Figure 9** and **Supplementary Table S2**). The expression of 1 gene of farnesyl diphosphate synthase (*FDPS*) that catalyzes isopentenyl diphosphate to form farnesyl diphosphate was upregulated. Two genes for farnesal dehydrogenase (*FALDH1* and *FALDH2*), an enzyme that converts farnesal to farnesoic acid, were upregulated. One gene for juvenile hormone acid methyltransferase (*JHAMT*), which converts juvenile hormone acid to JH, was upregulated. In this study, most DEGs in the JH synthesis pathway were upregulated, indicating that JH synthesis in *H. cunea* larvae was induced to increase after SM1 infection.

## 4 Discussion

In our study, the metabolic pathways affected by SM1 infection can be divided into 8 categories: energy metabolism, metabolism of cofactors and vitamins, amino acid metabolism, xenobiotic biodegradation and metabolism, nucleotide metabolism, carbohydrate metabolism, lipid metabolism, and metabolism of terpenoids and polyketides. Oxidative phosphorylation is an important pathway in energy metabolism and the main source of energy in organisms: it can produce ATP and provide energy for various life activities of organisms (Ruiz-Pesini et al., 2004; D’Elia et al., 2006). In this study, 13 genes in the oxidative phosphorylation pathway of *H. cunea* were downregulated after SM1 infection. In *Epiphyas postvittana* (Walker), some genes in the oxidative phosphorylation pathway were downregulated after infection with NPV (Gatehouse et al., 2007). A similar situation was observed in a *Musca domestica* Linnaeus study, after 48 h of bacterial infection, the genes related to oxidative phosphorylation were downregulated, indicating that energy homeostasis and mitochondrial function were disrupted in the late stage of infection (Tang et al., 2014). In *Ceracris kiangsu* Tsai, when oxidative phosphorylation was destroyed, the production of ATP was blocked, resulting in an imbalance between the supply and demand of energy metabolism and the failure to maintain the normal life activities of cells, ultimately leading to death (Zhao et al., 2004). Amino acids are important molecules in every organism, and various amino acids play different functions in the organism. Huang et al. (2009) found that after *Bombyx mori* (Linnaeus) was infected with *Bacillus bombyseptieus* Hartman, most genes related to metabolic pathways, including amino acid metabolism, were upregulated. This was because *B. mori* needed to meet the basic material and energy requirements for the growth and reproduction of *B. bombyseptieus* during infection. On the one hand, amino acids may be directly used by pathogens; on the other hand, when the body needs a large amount of energy, amino acids can be used as materials for energy supply, such as proline (Auerswald et al., 1998; Scaraffia and Wells, 2003). In addition, it may be related to the involvement of amino acids in the production of immune-related substances or cells (Xu et al., 2015). However, in our results, approximately half of the DEGs related to amino acid metabolism pathways were downregulated. We speculate that this situation may be related to the infection time and proliferation of the pathogen. With the aggravation of pathogen infection, the host is more severely damaged, and the proportion of upregulation and downregulation in amino acid metabolism pathway genes may gradually change. This needs to be verified in our future studies. According to our current results, SM1 infection caused metabolic dysfunction in larvae of *H. cunea.*

Cellular immunity is a defense response mediated by blood cells (hemocytes), including phagocytosis, encapsulation, nodulation, etc (Ali Mohammadie Kojour et al., 2020). The cellular uptake of particulates (>0.5 μ) within a plasma-membrane envelope is called phagocytosis, which is closely related to and partly overlaps the endocytosis of soluble ligands by fluid-phase macropinocytic and receptor pathways (Gordon, 2016). Actin can interact with many proteins and participate in many important biological processes, including immunity (Pollard and Cooper, 2009; Dominguez & Holmes, 2011). Phagocytosis is associated with the reorganization of the actin cytoskeleton, and actin can mediate phagocytosis and direct killing of bacteria (May and Machesky, 2001; Sandiford et al., 2015). When *Scylla paramamosain* Estampador lacked actin, they had higher morbidity and mortality after infection with *Vibrio alginolyticus* (Miyamoto et al.) Sakazaki or white spot syndrome virus (WSSV) (Sun et al., 2017b). V-ATPase is a class of important transporters that play an important role in plasma membrane proton transport in various cell types and are important for cellular processes (Wagner et al., 2004; Beyenbach and Wieczorek, 2006; Forgac, 2007; Hinton et al., 2009). Studies on many insects, including *D. citri*, *Sphenophorus levis* Vaurie, and *Locusta migratoria* (Linnaeus), have found that the interference of *V-ATPase* gene will affect the growth and survival of insects (Mohan et al., 2021; Guo et al., 2022; Liu et al., 2022). Studies have shown that RNAi knockdown of *V-ATPase* in *Monochamus alternatus* Hope inhibited the expression of immunity-related genes, including *lysozyme*, *TAK1*, and *pelle* (Li et al., 2022). In our results, there were downregulated genes related to the phagocytosis pathway in both *actin* and *V-ATPase*. This suggested that the relevant immune response was affected by SM1. Cathepsin is associated with various physiological processes such as immunity, aging, development and tissue remodeling (Conus, 2010; Stoka et al., 2016; Vidak et al., 2019), and its inadequate processing or activation can lead to cell death (Chwieralski et al., 2006). In studies of *B. mori* and *Procambarus clarkii* (Girard), CTSL was an important protease involved in the innate immune response (Dai et al., 2017; Sun et al., 2021). *CTSL* in the hepatopancreas of *Litopenaeus vannamei* (Boone) was downregulated after infection with WSSV (Zhai et al., 2021). CTSD has also been found to be associated with immune responses in invertebrates (Li et al., 2010; Menike et al., 2013; Yu et al., 2020). After knockout of *CTSD* from *Eriocheir sinensis* (H. Milne Edwards) with RNAi, the expression levels of many immune-related genes were decreased, and the mortality was increased after infection with *Spiroplasma eriocheiris* Wang (Ning et al., 2018). In this study, both *CTSL* and *CTSD* genes were downregulated after SM1 infection. The *CTSL* gene was not only located in the apoptosis, autophagy, and lysosome pathways similar to *CTSD* but also involved in the phagosome pathway. This suggested that these pathways were affected to some extent by SM1 infection. Lysosomes are the main degradation regions in cells. Lysosomes obtain substrates through phagocytosis, endocytosis, and autophagy and degrade them (Sagné et al., 2001; Saftig and Klumperman, 2009; Rong et al., 2011; Jézégou et al., 2012; Liu et al., 2012). In this study, in addition to *CTSL* and *CTSD*, downregulated genes in the lysosome pathway also included *CD53*, *CD63*, *NPC2* and *SMPD*. In studies of other arthropods, these genes have indeed been classified as immune-related genes (Wang et al., 2019; Lawrie et al., 2020; Liao et al., 2020). Studies have reported that stromal membrane-associated protein is involved in the regulation of endocytosis (Tanabe et al., 2006), and its gene was downregulated in our study. In conclusion, the cellular immune response of *H. cunea* larvae was suppressed or destroyed by SM1 infection.

Melanization is an important physiological process in insects that plays an important role in wound healing, cuticle tanning, immunity and so on (Nakhleh et al., 2017). Tryp and TLP are important enzymes in the process of melanization, and they have been reported to participate in immune defense reactions in a variety of animals (Zhou et al., 2012; Sun et al., 2017a; Yang et al., 2021). In *E. sinensis*, when *Tryp* was silenced, the expression of prophenoloxidase was downregulated, and the mortality rate of *E. sinensis* infected by *S. eriocheiris* increased (Gao et al., 2019). In this study, we found that 1 Tryp gene and 8 TLP genes were downregulated. In addition, 2 SPs genes with the Tryp domain were also downregulated. Obviously, SM1 inhibited the melanization of *H. cunea.* Cuticle proteins are important components of insect cuticles (Suderman et al., 2006; Arakane et al., 2012; Noh et al., 2016). Loss of cuticle protein genes blocks the deposition of melanin (Xiong et al., 2017). It has been found that the degree of melanism in silkworm larvae is positively correlated with the expression of cuticle protein genes (Qiao et al., 2020). In our results, two cuticle protein genes were downregulated, which obviously has a negative effect on melanization. Some studies have found that ACE and SPn are both negative regulators of melanization in different insects (Tong et al., 2005; Chu et al., 2015; Huybrechts and Coltura, 2018; Kausar et al., 2018). In this study, some genes in these negative regulators were upregulated. This represents an inhibition of melanism. In conclusion, our results indicated that SM1 infection inhibited the melanization in *H. cunea* larvae.

GSTs are a class of enzymes widely present in aerobic organisms. GSTs play an important role in the detoxification of exogenous and endogenous substances and are also involved in a variety of life functions (Enayati et al., 2005; Li et al., 2007). Insect CYPs function in several aspects, including feeding, growth, development, and protection against xenobiotics. Protection against xenobiotics includes resistance to pesticides and tolerance to plant toxins (Scott and Wen, 2001). When insects are infected with pathogens, detoxification enzymes might be inhibited. For example, studies have found that CYP activity in *Dysdercus koenigii* (Fabricius) was decreased after infection with *Aspergillus niger* van Tieghem, and a large number of GSTs in *Drosophila melanogaster* Meigen were downregulated in the overall response to fungi (Trienens et al., 2017; Kumar et al., 2019). Sun et al. (2019) found that different amounts of GST genes were inhibited in *H. cunea* larvae by the stress of different concentrations of HcNPV. Similar results were also found in our study, in which 3 *CYP* genes and 3 *GST* genes in *H. cunea* were downregulated by SM1 infection. This indicated that SM1 infection caused the inhibition of *CYPs* and *GSTs*, which would reduce the ability of *H. cunea* to metabolize various harmful substances including the metabolites of SM1.

When pathogens infect insects, immune-related genes are induced to improve the immune ability of insects to fight the pathogens. SM1 is a gram-negative bacteria. Lipopolysaccharide (LPS), a component of the cell wall of SM1, was a possible proinflammatory cytokine stimulant that could greatly induce the immune-related gene expression in some insects (Chappell et al., 2000). Therefore, we have reported that SM1 can induce the immunity of *H. cunea*, which upregulates many immune genes in *H. cunea* (Feng et al., 2021; Wang et al., 2021a). On the other hand, studies have shown that LPS was an important virulence factor of many pathogenic bacteria (Whitfield and Trent, 2014), which could help bacteria resist the active component of the host innate immune response and promote the survival of bacteria in the body; at the same time, it had the characteristics of endotoxin, causing cytokine storm and disease, and even leading to the death of the host (Anwar and Choi, 2014; Feng et al., 2023). However, there is no report at home and abroad about how SM1 destroys *H. cunea*. Therefore, we found that the immunity of *H. cunea* was inhibited after SM1 infection in this study. This study is of great significance to further study the interaction between SM1 and *H. cunea.*

JH is associated with the regulation of insect molting and metamorphosis and is an important hormone related to insect growth and development. In addition, JH also influences ovarian development, caste differentiation, orchestrating phase polyphenism, reproductive behavior and many other aspects (Hartfelder, 2000). In our study, most of the genes related to the JH synthesis pathway were upregulated after the *H. cunea* larvae were infected with SM1. This has also been found in studies involving *B. mori*. A study found that the expression of many JH-related genes was upregulated after *B. mori* infection with NPV and *B. bombyseptieus*, and it was speculated that the pathogens could prolong the larval period of hosts by activating JH-related genes to facilitate the pathogens to obtain more nutrients for reproduction (Huang et al., 2009). In the case of *S. exigua* and *S. litura*, studies have also found that JH analogue (JHA) treatment of host larva would extend the larval period, improve the food conversion rate and intake, and ultimately improve the pathogen proliferation efficiency and yield by improving the nutritional level of host larva (Liu et al., 2005; Lasa et al., 2007; Li et al., 2009). On the other hand, studies have shown that JH is also involved in insect immunity. Flatt et al. (2008) found that JH was an immunosuppressant that reduces the expression of antimicrobial peptide (AMP) genes in *D. melanogaster*. A similar situation was also found in *Aedes aegypti* (Linnaeus) (Chang et al., 2021). Studies have found that treatment with JH or JHA for *Eurygaster integriceps* Puton, *Neobellieria bullata* (Parker) and *G. mellonella* could reduce the number of hemocytes and inhibit the formation of nodules (Franssens et al., 2006; Zibaee et al., 2012; Sezer and Ozalp, 2015). In addition, JH was found to inhibit spreeding of plasmatocytes in *S. exigua* (Kim et al., 2008). In conclusion, the increase in JH not only benefits the proliferation of pathogens in terms of nutrition but also inhibits the immune function of insects. Our results showed that SM1 infection induced the upregulation of genes related to JH synthesis in *H. cunea*, which was detrimental to *H. cunea.*

## 5 Conclusions

In this study, we analyzed the metabolic, immune and hormonal damage caused by SM1 in *H. cunea* larvae using full-length SMRT transcriptome sequencing. In metabolic pathways, represented by amino acid metabolism and oxidative phosphorylation, many genes in the pathways were downregulated. In immunity, cellular immunity, melanism and detoxification enzymes were negatively affected to some extent. Among hormones, genes related to the juvenile hormone synthesis pathway were upregulate, which was unfavorable for *H. cunea* larvae. This study revealed the transcriptomic response of *H. cunea* to the infection of SM1 and provided theoretical support and a basis for the improvement of the control effect of SM1 and the biological control of *H. cunea*.

## Data availability statement

The datasets presented in this study can be found in online repositories. The names of the repository/repositories and accession number(s) can be found below: NCBI as SRR22263802.

## Author contributions

Conceptualization, FT. Methodology, LZ, FT and ZW. Software, LZ. Validation, LZ and ZW. Formal analysis, LZ and XT. Investigation, LZ and XT. Resources, FT. Data curation, LZ, FT, XT and ZW, Writing-original draft, LZ. Writing-review and editing, FT. Visualization, LZ. Supervision, FT. Project administration, FT. Funding acquisition, FT. All authors contributed to the article and approved the submitted version.
